# Gallic Acid Inhibits Mesaconitine-Activated TRPV1-Channel-Induced Cardiotoxicity

**DOI:** 10.1155/2022/5731372

**Published:** 2022-04-13

**Authors:** Shu Han, Liyuan Bao, Weifei Li, Kaiyang Liu, Ya'nan Tang, Xitao Han, Ziqin Liu, Hongyue Wang, Fengting Zhang, Shuo Mi, Hong Du

**Affiliations:** ^1^School of Chinese Materia Medica, Beijing University of Chinese Medicine, Beijing 102488, China; ^2^China Beijing Tong Ren Tang (Group) Co., Ltd., Beijing 100062, China

## Abstract

*Aconiti Kusnezoffii Radix* (Caowu) is often combined or processed with *Chebulae Fructus* (Hezi) to achieve attenuation purposes in Mongolian medicine. Mesaconitine (MA), a main bioactive ingredient of Caowu, is also famous for its high cardiotoxicity while exerting good anti-inflammatory and analgesic properties. Gallic acid (GA), one of the leading chemical components in Hezi, possesses cardiac protection. This study aimed to clarify the detoxification effects of GA from Hezi on MA-induced cardiotoxicity and whether the detoxification mechanism is related to the TRPV1 channel. Cell viability was determined by methyl thiazol tetrazolium (MTT), and lactate dehydrogenase (LDH) leakage rate was determined by ELISA. Hoechst 33258, JC-1, DCFH-DA, and Fluo-3 AM staining were conducted to detect apoptosis, mitochondrial membrane potential, reactive oxygen species (ROS), and Ca^2+^ respectively; TRPV1 channel current was recorded by whole-cell patch-clamp technology to observe the effect of GA and MA alone or in combination on TRPV1 channel. The results showed that GA exhibited pronounced detoxification effects on MA-induced cardiotoxicity. GA significantly inhibited the MA-induced decrease in cell viability; suppressed the MA-induced LDH leakage rate, apoptosis, and the release of ROS and Ca^2+^; and alleviated the reduction of mitochondrial membrane potential. We found that MA-induced cardiotoxicity was significantly attenuated in H9c2 cells pretreated with the TRPV1 antagonist BCTC. In the whole-cell patch-clamp experiment, the TRPV1 channel current increase was caused by the GA and MA treatment, whereas it was reduced by the cotreatment of GA and MA. Our data demonstrate that GA in Hezi can reduce MA-induced cardiotoxicity by inhibiting intracellular Ca^2+^ influx, restoring mitochondrial membrane potential, and reducing apoptosis. The detoxification mechanism may be related to the desensitization of the TRPV1 channel by the combined application of MA and GA.

## 1. Introduction

Aconiti *k*usnezoffii *Radix* (Caowu), the root of *Aconitum kusnezoffii* Reichb., has been widely used in clinical practice in traditional Chinese medicine (TCM) as well as in Mongolian and Tibetan medicine for treating rheumatoid arthritis, joint pain, rheumatic fever, cancer, and other symptoms for thousands of years [1–6]. Caowu has both medicinal value and high toxicity. Down the ages, various methods, including decoction, compatibility, and processing, were developed to ensure the ideal balance of safety and effectiveness [7, 8]. However, in the clinic, Caowu-induced poisoning still occurs from time to time because of the narrow therapeutic index [9–11].

In Mongolian medicine, Caowu is often combined or processed with Chebulae Fructus (Hezi) to achieve attenuation purposes [12, 13]. Hezi, the fruits of *Terminalia chebula* Retz. and *Terminalia chebula* Retz, var. *tomentella* Kurt, is an ethnodrug that has long been used in the Mongolian area of China. Local clinical evidence has indicated that Hezi has preventative effects on toxicity from Caowu [14]. Our previous research further supported this view because we have observed the acute toxicity of the raw Caowu and Hezi-processed Caowu on mice, also examined the effects on the survival rate of H9c2 cells, and found that the toxicity of Hezi-processed Caowu is significantly lower than that of raw Caowu [15, 16]. However, just as the mechanism of Caowu poisoning is still ambiguous, until now, there is a lack of explanations on Hezi relieving the toxicity of Caowu.

The principal active ingredients in Caowu are alkaloids with a C_19_-diterpenoid skeleton, including aconitine (AC), mesaconitine (MA), and hypaconitine (HA) [17]. MA is the most abundant and representative component of alkaloids in Caowu [6]. Despite the therapeutic benefits, MA is one of the well-known cardiotoxins, which can cause lethal arrhythmias [18–20].

Gallic acid (GA) is one of the main chemical components in Hezi [21]. GA possesses multiple pharmacological potentials, such as anti-inflammatory, anti-oxidant, anti-viral, cardiac protection, and so on [22, 23]. It has been proven that GA can alleviate arrhythmias induced by aconitine, but the specific detoxification mechanism needs to be further studied [24].

Transient receptor potential vanilloid-1 (TRPV1), a calcium ion channel activated by the botanical irritant capsaicin, endovanilloids, H^+^, organic acids, and temperature 42°C [25–27], has been implicated as a potential mediator of cardiomyocytes apoptosis because the direct activation of TRPV1 channel on the cardiomyocytes increases [Ca^2+^]i, enhances the superoxide production by mitochondria, and reduces the mitochondrial membrane potential [28–31]. Some studies have also suggested that TRPVs are potential molecular targets for treating *Aconitum* species poisoning [32–35]. However, whether the TRPV1 channel and the precise molecular mechanism mediate the protective effect of Hezi against Caowu poisoning remains to be elucidated.

Therefore, in this study, we evaluated the detoxification effects of GA from Hezi on MA-induced H9c2 cells injury, and the detoxification mechanism by regulating the TRPV1 channel was explored in detail. We investigated the changes in H9c2 cells viability by methyl thiazol tetrazolium bromide (MTT) assay and measured the lactate dehydrogenase (LDH) leakage. ELISA was used to elucidate further the mediation of nuclear, intracellular calcium ions, intracellular ROS, and mitochondrial membrane potential. Besides, TRPV1 current was recorded by whole-cell patch-clamp technology, as a direct observe the effect of GA and MA alone or in combination on the TRPV1 channel.

## 2. Materials and Methods

### 2.1. Materials

Mesaconitine (molecular formula: C_33_H_45_NO_11_; purity >99.8%) and gallic acid (molecular formula: C_7_H_6_O_5_; purity >99.8%) were obtained from Chengdu DeSiTe Biotechnology Co. Ltd. (Sichuan, China). BCTC (molecular formula: C_20_H_25_ClN_4_O; purity ≥98%) was purchased from AbMole (AbMole, Houston, United States). All the compounds above were dissolved in dimethyl sulfoxide (DMSO, Sigma, USA) as a stock solution, sonicated for 10 min, and then diluted in Dulbecco's modified eagle's medium (DMEM; Lablead, Beijing, China) to the corresponding concentration when used. The final concentration of DMSO was lower than 0.1% (v/v).

### 2.2. Cell Cultures and Experimental Grouping

H9c2 cells (Cell Resource Center, IBMS, CAMS/PUMC, Beijing, China) were cultivated in DMEM supplemented with 10% fetal bovine serum (FBS) and 1% (v/v) penicillin/streptomycin solution in a humidified incubator at 37°C in a 5% carbon dioxide (CO_2_) atmosphere. When growing to the logarithmic phase, cells are either passaged or seeded (80∼90% cell density).

Experimental grouping: (1) control group: H9c2 cells were cultured for 24 h without any treatment; (2) MA group: H9c2 cells were treated with 250 µM MA for 24 h; (3) BCTC + MA group: H9c2 cells were pretreated with BCTC (10 µM) for 30 min before 250 µM MA; and (4) GA + MA group: H9c2 cells were treated with 250 µM MA and different concentrations of GA (25, 50, and 100 µM) for 24 h.

### 2.3. Cell Viability and LDH Leakage Rate Assays

Cell viability and LDH leakage rate were determined by MTT assay (Beyotime Institute of Biotechnology, Shanghai, China) and measured the LDH release (Beyotime Institute of Biotechnology, Shanghai, China), respectively. Briefly, H9c2 cells were seeded at a density of 1 × 10^4^ cells per well in 96-well plates and incubated in an incubator at 37°C with 5% CO_2_ for 24 h. After treatment as described in Section 2.2, 100 µL MTT (0.5 mg/mL) solution was added, before incubation for 4 h in darkness; then 150 µL DMSO was used to lyse MTT formazan. Each well's optical density (OD) was measured at 490 nm using a microplate reader (BMG Labtech, Offenburg, Germany). LDH leakage rate from cell supernatants was measured using the LDH cytotoxicity kit according to the manufacturer's instructions. The optical density of each well was measured with a microplate reader (BMG Labtech, Offenburg, Germany) at a wavelength of 490 nm, and the LDH leakage rate (%) was calculated.

### 2.4. Apoptosis Morphology Assay

Hoechst 33258 kit (Beyotime Institute of Biotechnology, Shanghai, China) was used to determine the morphological changes of apoptosis. Briefly, H9c2 cells were seeded in 24 well plates at a density of 5 × 10^4^ cells/well and incubated for 24 h. According to the manufacturer's instruction after treatment as described in Section 2.2, the images of nuclear morphological changes in the cells were taken under the Olympus fluorescence microscope (magnification, ×100, Nikon, Japan) at a 461 nm emission. The data were analyzed to represent the mean fluorescence intensity (MFI) with ImageJ software.

### 2.5. Intracellular ROS Assessment

Detection of intracellular ROS was performed using a dichloro-dihydro-fluorescein diacetate (DCFH-DA) kit (Beyotime Institute of Biotechnology, Shanghai, China). In brief, after H9c2 cells were treated as described in Section 2.2, the medium was removed, added 10 µM DCFH-DA, and incubated for 30 min at 37°C. Subsequently, the cells were washed with a serum-free medium, and the images were taken under the fluorescence microscope (magnification, ×100, Nikon, Japan). The data were analyzed to represent the MFI with ImageJ software.

### 2.6. Mitochondrial Membrane Potential Measurement

The mitochondrial membrane potential changes in H9c2 cells were determined by JC-1 kit (Beyotime Institute of Biotechnology, Shanghai, China). In short, H9c2 cells were treated as described in Section 2.2, the H9c2 cells were incubated with JC-1 staining solution (10 µg/mL) for 20 min at 37°C in the dark and rinsed twice with PBS, and the images were taken under the fluorescence microscope (magnification, ×100, Nikon, Japan). The change of mitochondrial membrane potential was reflected by the ratio of red fluorescence to green fluorescence.

### 2.7. Intracellular Ca^2+^ Production Measurement

Changes in intracellular Ca^2+^ release were detected using the Fluo-3 AM kit (Beyotime Institute of Biotechnology, Shanghai, China). After treatment as described in Section 2.2, the H9c2 cells were incubated, loaded with a medium containing 5 µM Fluo-3AM (500 µL/well) for 60 min at 37°C in the dark, and rinsed with PBS; the images were taken under the fluorescence microscope (magnification, ×100, Nikon, Japan). The data were analyzed to represent the MFI with ImageJ software. Mean fluorescence intensity was used to evaluate the extent of Ca^2+^ efflux.

### 2.8. Whole-Cell Patch-Clamp Recording

TRPV1-HEK293 cells were cultured in DMEM medium supplemented with 10% FBS, 100 µg/mL Zeocin and 10 µg/mL Blastincidin in the culture dish. Cells grew in a humidified incubator at 37°C with 5% CO_2_. For the manual patch-clamp test, the cells were detached using the TrypLE™ Express solution. Then 8 × 10^3^ cells were seeded into a 24-well plate (final medium volume: 500 *μ*L) with 1 coverslip in each well. The current was induced by tetracycline for 18 h. All cell culture procedures followed the cell culture SOP of ICE Bioscience Inc. TRPV1 receptor current was recorded at a holding potential of −70 mV with gap-free mode to record the peak current after the test article application from low to high concentrations. The brief experimental protocol is described as follows: the 10 µΜ Capsaicin and each concentration of GA and MA alone or in combination will be applied 1–2 times followed by a 1 min wash-in using the extracellular solution. The next test concentration will be tested.

The data will be collected by EPC-10 amplifier and stored in PatchMaster (HEKA) soft. Glass pipette was prepared with a micropipette puller. The glass pipette was manipulated using a micromanipulator under the microscope. After touching the cell, a slight suction was applied to achieve high seal resistance (GΩ). Fast capacitance (in pF) compensation was made after achieving a high seal, and the membrane was broken. After the whole-cell mode was achieved, cell capacitance (in pF) compensation was made from whole-cell capacitance compensation. No leak subtraction was made. The test and control solutions have flowed into a recording chamber mounted on the stage of an inverted microscope via a gravity-fed solution delivery system. Solution solutions were withdrawn from the recording chamber by vacuum aspiration during the experiment. Each concentration was tested multiple times. All the tests were performed at room temperature. The current value was standardized through the whole-cell capacitance and was shown using pA/pF.

### 2.9. Statistical Analysis

The experimental data were analyzed using GraphPad Prism software version 8.0.1 (GraphPad Software, Inc. La Jolla, USA) and ImageJ 1.8.0 (Bethesda, Maryland, USA). Statistically significant differences were performed through one-way analysis of variance (ANOVA), and Sidak test was used for pairwise comparison. The results were expressed as the mean ± standard deviation (SD). *P* < 0.05 was considered statistically significant.

## 3. Results

### 3.1. MA Inhibited Cell Viability in H9c2 Cells

**Figure 1:**
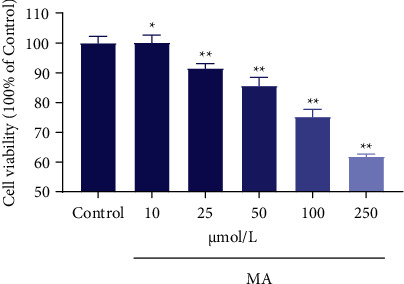
H9c2 cells were treated with various concentrations of MA for 24 h. Cell viability was quantified and expressed as a percentage of the control group. The viability of the control group was defined as 100%. Data are presented as the mean ± SD (*n* = 3). ^*∗*^*P* < 0.05 versus control group and ^*∗∗*^*P* < 0.01 versus control group.

As shown in Figure 1, 0–250 µM MA treatment for 24 h affected the proliferation of H9c2 cells to varying degrees. Compared with the control group, 25–250 µM MA reduced the survival rate of H9c2 cells in a concentration-dependent manner, especially at 250 µM, cell viability was significantly decreased to 61.88 ± 0.78%, indicating that MA could significantly inhibit the proliferation of H9c2 cells in the range of 25–250 µM. To further elucidate the toxic effect and mechanism of MA on H9c2 cells, 250 µM of MA was chosen in subsequent experiments.

### 3.2. GA Treatment Improved MA-Induced Cell Viability and Inhibited LDH Leakage Rate

**Figure 2:**
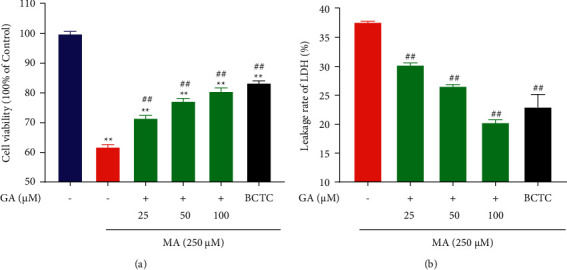
Effect of GA and the coadministrative of MA on the cell viability and LDH leakage rate. (a) Effects of GA (25, 50, and 100 µM) or pretreatment with BCTC (10 µM) on the decrease of cell viability in H9c2 cells induced by 250 µM MA. The viability of the control group was defined as 100%. (b) Effects of GA (25, 50, and 100 µM) or pretreatment with BCTC (10 µM) on the increase of LDH leakage rate in H9c2 cells induced by 250 µM MA. Data are presented as the mean ± SD (*n* = 3). ^*∗∗*^*P* < 0.01 versus control group, and ^##^*P* < 0.01 versus MA group.

Cell survival rate and LDH leakage rate were generally used as indicators of cytotoxicity [36]. To investigate the effect of GA on H9c2 cells toxicity induced by MA, the survival rate of H9c2 cells was measured. As shown in Figure 2(a), the results show that 250 µM MA significantly decreased the cell viability of H9c2 cells, and the survival rate was reduced to 61.85 ± 0.74% (*P* < 0.01). Treatment with GA at concentrations of 25, 50, and 100 µM significantly suppressed the decrease of cell viability in a concentration-dependent manner (*P* < 0.01), indicating that GA increased cell viability and protected cardiomyocytes against MA-induced injury. LDH is a stable enzyme expressed in the cytoplasm of myocardial cells [37]. When the cells are damaged, the cell membrane will be destroyed, and a large amount of LDH will be released. Therefore, the content of LDH in the cell supernatant can indirectly reflect the degree of cell damage. As shown in Figure 2(b), H9c2 cells could significantly promote the release of LDH in the cells after incubating with 250 µM MA. However, it can be blocked by 25, 50, and 100 µM GA, and the LDH leakage rate gradually decreases with the increase of concentration (*P* < 0.01).

To investigate the role of the TRPV1 channel in MA-induced H9c2 cells, we added BCTC, a TRPV1 channel antagonist [38]. Pretreatment with BCTC (i.e., TRPV1 channel blocking) significantly increased the viability of H9c2 cells and inhibited the leakage rate of LDH (*P* < 0.01). Our data showed that MA might exert some toxicity by mediating the TRPV1 channel.

### 3.3. GA Treatment Inhibited MA-Induced Apoptosis and ROS Release

**Figure 3:**
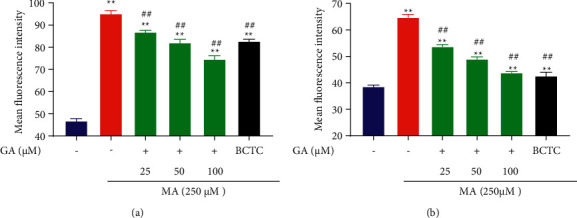
Effect of GA and the coadministrative of MA on the mean fluorescence intensity of nucleus and ROS in H9c2 cells. (a) Effects of GA (25, 50, and 100 µM) or pretreatment with BCTC (10 µM) on the increase of the mean fluorescence intensity of the nucleus in H9c2 cells induced by 250 µM MA. (b) Effects of GA (25, 50, and 100 µM) or pretreatment with BCTC (10 µM) on the increase of the mean fluorescence intensity of ROS in H9c2 cells induced by 250 µM MA. Data are presented as the mean ± SD (*n* = 3). ^*∗∗*^*P* < 0.01 versus control group and ^##^*P* < 0.01 versus MA group.

**Figure 4:**
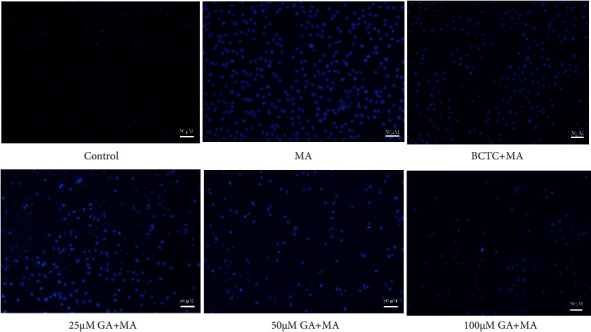
Effect of GA and the coadministrative of MA on the apoptosis in H9c2 cells. H9c2 cells were incubated with GA (25, 50, and 100 µM) or pretreated with BCTC (10 µM) in H9c2 cells induced by 250 µM MA. Nucleus fluorescence images are visualized by a fluorescence microscope (magnification, ×100; scale bar, 50 µm).

**Figure 5:**
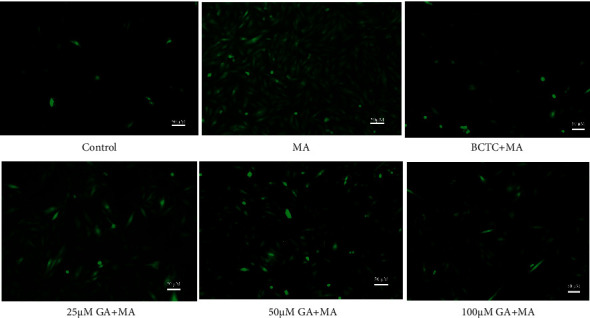
Effect of GA and the coadministrative of MA on the ROS in H9c2 cells. H9c2 cells were incubated with GA (25, 50, and 100 µM) or pretreated with BCTC (10 µM) in H9c2 cells induced by 250 µM MA. ROS fluorescence images are visualized by a fluorescence microscope (magnification, ×100; scale bar, 50 µm).

The overproduction of ROS generally accompanies the occurrence of cardiotoxicity, and the accumulation of ROS can induce the generation of oxidative stress in cardiomyocytes and then induce the occurrence of apoptosis to a certain extent [39, 40]. To further explore the effect of GA on apoptosis and ROS in MA-induced H9c2 cells, we used DCFH-DA and Hoechst 33258 to detect the MA-induced ROS production and nuclear morphology changes in H9c2 cells, respectively. As shown in Figures 3(a) and 4, the nucleus of the control group showed uniform blue fluorescence. Many apoptotic cells appeared after being treated with 250 *μ*M MA. The nucleus chromatin was pyknotic, showing bright blue fluorescence of dense concentration staining (*P* < 0.01). When the TRPV1 channel was blocked, the mean fluorescence intensity of the nucleus could be significantly reduced, and the damaged state of the nucleus could be improved (*P* < 0.01). After treatment with GA at a concentration of 25, 50, and 100 *μ*M, it was found that the pyknosis of nuclear chromatin was significantly weakened, and the intensity of bright blue fluorescence of dense concentration staining gradually decreased with the increase of concentration (*P* < 0.01). As shown in Figures 3(b) and 5, compared with the control group, the green fluorescence of H9c2 cells treated with 250 *μ*M MA was significantly increased; that is, the level of ROS in the cells increased (*P* < 0.01). When the TRPV1 channel was blocked, green fluorescence intensity was significantly attenuated, and ROS release was reduced (*P* < 0.01). After 24 h of treatment with GA (25, 50, and 100 *μ*M), the green fluorescence intensity in the cells was lower than that of 250 *μ*M MA in a concentration-dependent manner (*P* < 0.01).

### 3.4. GA Treatment Alleviated MA-Induced Mitochondrial Membrane Potential and Inhibited Ca^2+^ Release

**Figure 6:**
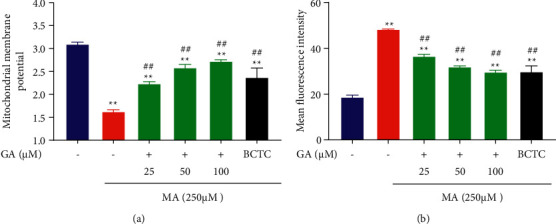
Effect of GA and the coadministrative of MA on the mitochondrial membrane potential and Ca^2+^ release in H9c2 cells. (a) Effects of GA (25, 50, and 100 µM) or pretreatment with BCTC (10 µM) on the decrease of the mitochondrial membrane potential in H9c2 cells induced by 250 µM MA. (b) Effects of GA (25, 50, and 100 µM) or pretreatment with BCTC (10 µM) on the increase of the mean fluorescence intensity of Ca^2+^ in H9c2 cells induced by 250 µM MA. Data are presented as the mean ± SD (*n* = 3). ^*∗∗*^*P* < 0.01 versus control group and ^##^*P* < 0.01 versus MA group.

**Figure 7:**
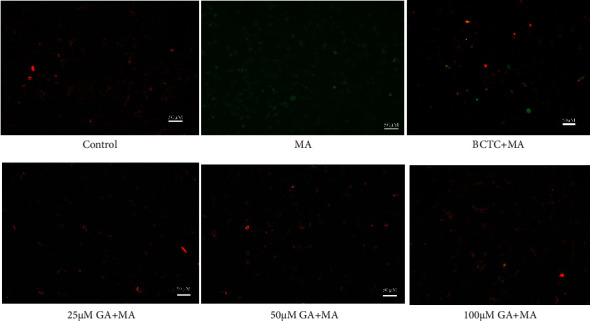
Effect of GA and the coadministrative of MA on the mitochondrial membrane potential of H9c2 cells. H9c2 cells were incubated with GA (25, 50, and 100 µM) or pretreated with BCTC (10 µM) in H9c2 cells induced by 250 µM MA. Mitochondrial membrane potential fluorescence images are visualized by a fluorescence microscope (magnification, ×100; scale bar, 50 µm).

**Figure 8:**
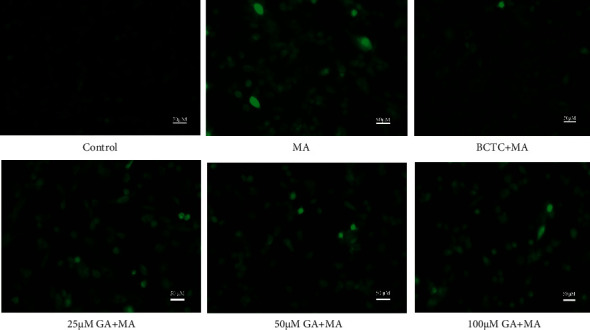
Effect of GA and the coadministrative of MA on the Ca^2+^ release in H9c2 cells. H9c2 cells were incubated with GA (25, 50, and 100 µM) or pretreated with BCTC (10 µM) in H9c2 cells induced by 250 µM MA. Ca^2+^ release fluorescence images are visualized by a fluorescence microscope (magnification, ×100; scale bar, 50 µm).

Literature studies show that mitochondria are not only the central organ of cell metabolism and cell respiration, but also regulate the intracellular Ca^2+^ concentration. When the mitochondrial function is dysfunctional or damaged, it will lead to continuous Na^+^ influx in the cell, activate Na^+−^Ca^2+^ exchange protein, increase the intracellular Ca^2+^ concentration, lead to intracellular calcium overload, and then lead to mitochondrial membrane depolarization, mitochondrial membrane potential reduction, and even cell death [41, 42]. Figures 6(a) and 7 show that the control group cells mostly showed red fluorescence and a small amount of green fluorescence, which indicated that the mitochondrial membrane potential was average. After treatment with 250 *μ*M MA, the red fluorescence intensity decreased, and the green fluorescence intensity increased significantly, which significantly reduced the mitochondrial membrane potential (*P* < 0.01), and increased mitochondrial membrane potential after TRPV1 channel blockade (*P* < 0.01). After treatment with GA at a concentration of 25, 50, and 100 *μ*M, the cells' red fluorescence/green fluorescence ratio was enhanced, and the mitochondrial membrane potential increased dose-dependent (*P* < 0.01). As shown in Figures 6(b) and 8, compared with the control group, the green fluorescence of H9c2 cells treated with 250 *μ*M MA was significantly increased, that is, the intracellular Ca^2+^ release increased significantly (*P* < 0.01); inhibiting the sustained Ca^2+^ influx when TRPV1 channel was blocked (*P* < 0.01). After treatment with GA (25, 50, and 100 *μ*M), the green fluorescence intensity in the cells decreased in a concentration-dependent manner (*P* < 0.01).

### 3.5. Effects of MA, GA, and the Combination of Both on TRPV1 Channel Current

**Figure 9:**
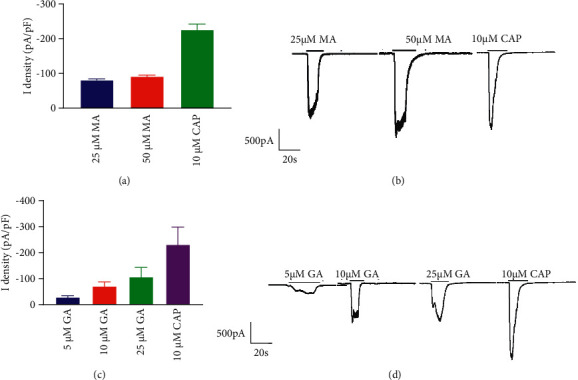
Current changes in TRPV1-HEK293 cells treated with MA and GA alone. (a) and (c) The bar graph shows the quantified data by calculating the current densities. (b) and (d) Representative traces of MA (25 and 50 µM) and GA (5, 10, and 25 µM) in TRPV1-HEK293 cells. The current densities are presented as the mean ± SD (*n* = 3). The holding potential used was −70 mV. CAP: capsaicin, MA: mesaconitine, and GA: gallic acid.

To further explore the mechanism by which GA alleviates MA-induced cardiotoxicity, we also evaluated the effects of MA, GA, and the combination of both on TRPV1 channel current by a whole-cell patch clamp. As shown in Figures 9(a) and 9(b), the TRPV1 current could be activated by 10 µM capsaicin, an agonist of the TRPV1 channel. When the concentration of MA was 25 µM, a significant TRPV1 channel current was generated with a current density of –80.68 ± 6.51, which increased to –91.53 ± 6.77 as the concentration increased. TRPV1 channel current was elicited when GA was present at a concentration of 5 µM; with the increase of concentration, the current increased significantly, as shown in Figures 9(c) and 9(d). It suggests that similar to capsaicin, both MA and GA can act as agonists for the TRPV1 channel.

**Figure 10:**
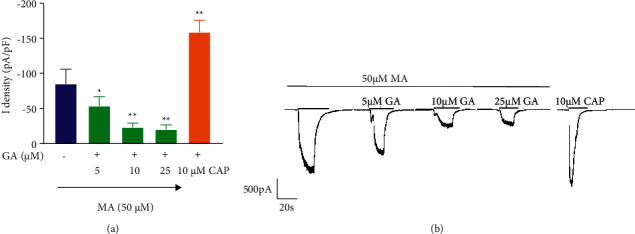
Changes of current in TRPV1-HEK293 cells cotreated with GA (5, 10, and 25 µM) and MA (50 µM). (a) The bar graph shows the quantified data by calculating the current densities. (b) GA (5, 10, and 25 *μ*M) and the co-administrative of 50 *μ*M MA on the representative traces in TRPV1-HEK293 cells. The current densities are presented as the mean ± SD (*n* = 3). ^*∗*^*P* < 0.05 versus MA group and ^*∗∗*^*P* < 0.01 versus MA group. The holding potential used was −70 mV.

It can be seen from Figure 10 that when MA and GA are applied in combination, 50 µM MA can produce a prominent TRPV1 channel current. Compared with the MA Group, 5 µM GA reduced the intensity of TRPV1 current (*P* < 0.05) and further inhibited the intensity of TRPV1 current with the increase of concentration (*P* < 0.01).

## 4. Discussion

Mesaconitine, a main active ingredient of Caowu, is notorious for its high cardiotoxicity and neurotoxicity, of which cardiotoxicity is the primary toxic reaction, which could result in arrhythmia, ventricular tachycardia (VT), ventricular fibrillation (VF), and even sudden death if used excessively [43–47]. Previous studies have demonstrated the mechanisms by which MA induces cardiotoxicity are as follows: (1) blocking the inactivation of voltage-dependent sodium channels and prolonging potential action durations (APDs) and (2) acetylcholine released by exciting the vagus nerve will directly inhibit the atrioventricular node or excite the ectopic pacemaker, thereby leading to arrhythmia [48–51].

The TRPV1 channel is a thermosensitive channel that can be activated by Aconitum plants and components such as capsaicin and aconitine, which in turn can induce apoptosis, Ca^2+^ overload, and so on, leading to a certain degree of toxicity [52–55]. Some literature also suggested that one of the causes of cardiotoxicity of Caowu may also be related to the activation of TRPV1 channel [31, 33, 34]. Meanwhile, our experiments also proved this notion: activation of TRPV1 channel induced by MA could accelerate the generation of apoptosis, decrease the mitochondrial membrane potential, and intracellular Ca^2+^ overload, which was significantly reversed by BCTC, a TRPV1 antagonist. All the above indicate that myocardial injury is significantly aggravated when TRPV1 channel is activated.

It was reported that the TRPV1 channel is subject to dose-dependent sensitization or desensitization, that is to say, low-dose agonists can sensitize the TRPV1 channel. On the other hand, the TRPV1 channel can be desensitized upon prolonged activation or repeated exposures to agonists [56, 57]. As a protective mechanism, desensitization of the TRPV1 channel can reduce the occurrence of cardiotoxicity by inhibiting a large amount of Ca^2+^ influx to some degree [58, 59], GA was found to reduce MA-induced cardiotoxicity by improving cell viability; inhibiting LDH, ROS, Ca^2+^, as well as apoptosis; and alleviating the reduction of mitochondrial membrane potential in this study, whether the exertion of the detoxification effect is also related to the desensitization mechanism deserves to be explored. To further elucidate this, we measured the TRPV1 channel activity by measuring TRPV1 channel current upon GA and MA treatment, respectively, and cotreatment in TRPV1-HEK293 cells. In TRPV1-HEK293 cells, the TRPV1 channel current increase was caused by the GA and MA treatment, whereas it was reduced by the cotreatment of GA and MA. Furthermore, as shown in the above studies, when MA is combined with GA, it can reduce the cardiotoxicity induced by MA. so this study speculated that the mechanism of action of GA to attenuate MA-induced cardiotoxicity might be due to the synergistic effect of MA and GA to desensitize TRPV1 channel to exert an attenuated effect partially.

TRPV1 channel is also a target for pain treatment, and its analgesic effect is mainly achieved by inactivating the TRPV1 channel or chronic desensitization [60]. Clinical studies have shown that *Aconitum* herbs can inhibit anti-inflammatory and analgesic effects, also related to the TRPV1 channel [32]. Our experiment found that the combination of gallic acid and mesaconitine could achieve a particular desensitization effect on TRPV1 channels to reduce cardiotoxicity by inhibiting the influx of calcium ions in cardiomyocytes. However, whether the desensitization caused by the combination of gallic acid and mesaconitine also has a specific analgesic effect needs to be verified by further experiments.

This study also had some limitations. On the one hand, although we found that GA, the main active component in Hezi, can reduce the cardiotoxicity induced by MA, the leading active ingredient in Caowu, whether other chemical components in Caowu and Hezi can also play a similar role needs to be further confirmed. On the other hand, this study was conducted only at the cellular level and lacked whole animal experiments to corroborate. It should not be ignored that some works of literature have also proved that the occurrence of arrhythmias, tachycardias, and other phenomena induced by *Aconitum* species can also be partially reversed in the presence of a large number of calcium ions [61], which also provides a basis for our subsequent studies, that is, whether it will also reverse the arrhythmia effect induced by Caowu after processing or compatibility with Hezi in the clinical use is a question worthy of discussion. This will also provide a basis for the safe and rational use of *Aconitum* species in the clinic.

## 5. Conclusions

The aforementioned results show that the detoxification of MA-induced cardiotoxicity by GA can be achieved via increasing cell ability, suppressing the release of LDH, ROS, Ca^2+^, and the occurrence of apoptosis, restoring mitochondrial membrane potential. In addition, this study also found that MA and GA could act as agonists for the TRPV1 channel similar to capsaicin, yet the TRPV1 channel current was reduced at the cotreatment of GA and MA. From this, we speculated that the detoxification of MA-induced cardiotoxicity by GA might be related to the desensitization of the TRPV1 channel by the combined application of MA and GA.

## Data Availability

The data used to support the findings of this study are available from the first author (hs361015@163.com) upon reasonable request.

## Abbreviations

MA: Mesaconitine
GA*:*: Gallic acid
LDH: Lactate dehydrogenase
MTT*:*: Methylthiazoltetrazolium bromide
ROS: Reactive oxygen species
TRPV1*:*: Transient receptor potential vanilloid-1
TCM*:*: Traditional Chinese medicine
AC*:*: Aconitine
HA*:*: Hypaconitine
DMSO: Dimethyl sulfoxide
DMEM: Dulbecco's modified eagle's medium
FBS: Fetal bovine serum
CO2: Carbon dioxide
OD: Optical density
MFI: Mean fluorescence intensity
DCFH-DA: Dichloro-dihydro-fluorescein diacetate
VT: Ventricular tachycardia
VF: Ventricular fibrillation
APDs: Action potential durations.
